# 4316 Preoperative Goal-setting by Patients is Correlated with Baseline and Not 6-week Outcomes following Total Knee Arthroplasty (TKA)

**DOI:** 10.1017/cts.2020.421

**Published:** 2020-07-29

**Authors:** Vesta Nwankwo, Janet Bettger, Cindy L. Green, Thomas Risoli

**Affiliations:** 1 Duke University

## Abstract

OBJECTIVES/GOALS: Patient beliefs and goals can facilitate discussion of recovery expectations, patient-provider collaboration and maximization of goal achievement. In this study, we sought to address an evidence gap and examine the association of preoperative self-assessment of goals with preoperative and 6-week knee function and gait speed among total knee arthroplasty (TKA) patients. METHODS/STUDY POPULATION: We conducted a secondary analysis of data from the VERITAS randomized, controlled trial conducted from 11/2016-03/2018 that included adults age ≥ 18 years with scheduled and completed unilateral TKA followed by post-surgical physical therapy. Patients rated their ability to perform various activities of daily living goals scaled from 0 (unable to perform) to 10 (full performance). Patients were categorized by pre-surgical (baseline) goal rating: low = 0-2, intermediate = 3-4, and high = 5-10. Outcomes including gait speed and the KOOS were assessed within 10 days prior to surgery and 6-weeks post-surgery. Descriptive statistics and outcomes were compared for patients by preoperative goal rating using Chi-square or Fisher’s exact tests and ANOVA or Kruskal-Wallis tests as appropriate. RESULTS/ANTICIPATED RESULTS: Of 288 patients (mean age 65±8; 62.5% women; 82% white), 102 had a low goal rating (GR), 86 intermediate, and 99 high. Patients with low GR preoperatively generally had lower baseline mean scores than intermediate and high GR patients, respectively, on the KOOS (33.9/35.6/39.8; p<0.001) and lower gait speed (m/s) compared to intermediate and high GR patients at baseline (0.9/1.1/1.0; p = 0.009). The low, intermediate, and high GR groups, respectively, showed no difference across mean KOOS scores (61.0/61.2/61.9; p = 0.63) or gait speed (m/s) (1.0/1.0/1.0, p = 0.33) at 6 weeks postoperative. DISCUSSION/SIGNIFICANCE OF IMPACT: In this study, adults who perceived greater difficulty with a pre-selected activity goal, exhibited lower function prior to TKA but showed no differences in function 6-weeks after surgery. Follow-up studies will describe the association between goal-setting preoperatively and patient goal attainment and satisfaction following surgery.

